# Peroxisomal Multifunctional Protein 2 Deficiency Perturbs Lipid Homeostasis in the Retina and Causes Visual Dysfunction in Mice

**DOI:** 10.3389/fcell.2021.632930

**Published:** 2021-02-02

**Authors:** Yannick Das, Daniëlle Swinkels, Sai Kocherlakota, Stefan Vinckier, Frédéric M. Vaz, Eric Wever, Antoine H. C. van Kampen, Bokkyoo Jun, Khanh V. Do, Lieve Moons, Nicolas G. Bazan, Paul P. Van Veldhoven, Myriam Baes

**Affiliations:** ^1^Laboratory of Cell Metabolism, Department of Pharmaceutical and Pharmacological Sciences, KU Leuven, Leuven, Belgium; ^2^Laboratory of Angiogenesis and Vascular Metabolism, Department of Oncology, KU Leuven–VIB, Leuven, Belgium; ^3^Laboratory of Genetic Metabolic Diseases, Department of Clinical Chemistry and Pediatrics, Amsterdam University Medical Center (UMC), University of Amsterdam, Amsterdam, Netherlands; ^4^Core Facility Metabolomics, Amsterdam University Medical Center (UMC), Amsterdam, Netherlands; ^5^Bioinformatics Laboratory, Department of Epidemiology and Data Science, Amsterdam Public Health Research Institute, Amsterdam University Medical Center (UMC), University of Amsterdam, Amsterdam, Netherlands; ^6^Biosystems Data Analysis, Swammerdam Institute for Life Sciences, University of Amsterdam, Amsterdam, Netherlands; ^7^Neuroscience Center of Excellence, School of Medicine, Louisiana State University Health New Orleans, New Orleans, LA, United States; ^8^Animal Physiology and Neurobiology, Department of Biology, KU Leuven, Leuven, Belgium; ^9^Lipid Biochemistry and Protein Interactions (LIPIT), Department of Cellular and Molecular Medicine, KU Leuven, Leuven, Belgium

**Keywords:** peroxisome, β-oxidation, multifunctional protein 2, retina, photoreceptors, PUFA, DHA

## Abstract

Patients lacking multifunctional protein 2 (MFP2), the central enzyme of the peroxisomal β-oxidation pathway, develop retinopathy. This pathway is involved in the metabolism of very long chain (VLCFAs) and polyunsaturated (PUFAs) fatty acids, which are enriched in the photoreceptor outer segments (POS). The molecular mechanisms underlying the retinopathy remain, however, elusive. Here, we report that mice with MFP2 inactivation display decreased retinal function already at the age of 3 weeks, which is accompanied by a profound shortening of the photoreceptor outer and inner segments, but with preserved photoreceptor ultrastructure. Furthermore, MFP2 deficient retinas exhibit severe changes in gene expression with downregulation of genes involved in the phototransduction pathway and upregulation of inflammation related genes. Lipid profiling of the mutant retinas revealed a profound reduction of DHA-containing phospholipids. This was likely due to a hampered systemic supply and retinal traffic of this PUFA, although we cannot exclude that the local defect of peroxisomal β-oxidation contributes to this DHA decrease. Moreover, very long chain PUFAs were also reduced, with the exception of those containing ≥ 34 carbons that accumulated. The latter suggests that there is an uncontrollable elongation of retinal PUFAs. In conclusion, our data reveal that intact peroxisomal β-oxidation is indispensable for retinal integrity, most likely by maintaining PUFA homeostasis.

## Introduction

Vision encompasses a well-orchestrated process that is initiated at the retina. To date, a plethora of mutations are recognized to cause developmental and degenerative retinal diseases. In many peroxisomal disorders, several compartments of the visual system are often severely affected (Folz and Trobe, 1991; Das and Baes, 2019). However, the underlying mechanisms still remain largely unresolved.

Peroxisomal disorders are a group of inherited metabolic diseases that can be subdivided into two categories: peroxisome biogenesis disorders (PBD), of which Zellweger syndrome is the most severe, and single enzyme deficiencies (SED) (Waterham et al., 2016). Mutations in the central enzyme of the peroxisomal β-oxidation pathway, the *D*-specific multifunctional protein 2 (MFP2) encoded by the *HSD17B4* gene, cause a diverse spectrum of clinical disease presentations. The most severe clinical presentation is indistinguishable from Zellweger syndrome, characterized by general multi-organ dysfunction, and is mostly fatal within the first year of life. These patients often develop retinopathy with reduced electroretinography (ERG) responses (Ferdinandusse et al., 2006; Argyriou et al., 2016). Interestingly, new and milder types of MFP2 deficiency with juvenile onset were recently described. Although visual acuity was normal, these patients presented with abnormal retinal pigmentation, whether or not coinciding with abnormal ERG responses (McMillan et al., 2012; Lines et al., 2014; Amor et al., 2016).

Peroxisomal β-oxidation executes the breakdown of various substrates, such as very long chain fatty acids (VLCFAs), the 2-methyl branched chain fatty acid pristanic acid, bile acid intermediates, and dicarboxylic acids. This pathway is also involved in both the catabolism and synthesis of poly-unsaturated fatty acids (PUFAs), such as docosahexaenoic acid (DHA, C22:6*n*-3) (Van Veldhoven, 2010). Interestingly, DHA is highly enriched in the photoreceptor outer segments (POS), a form of modified primary cilia where light is captured and transformed into an electrical signal (i.e., phototransduction). It is accepted that the diet and *de novo* hepatic biosynthesis, starting from α-linolenic acid (C18:3*n*-3), are the major retinal DHA sources, next to limited local synthesis (Bazan et al., 1982, 2011; Scott and Bazan, 1989; Wang and Anderson, 1993; Rotstein et al., 1996; Simon et al., 2016). Retinal DHA accretion includes a close collaboration between the photoreceptors and the retinal pigment epithelium (RPE), which is a monolayer of postmitotic cuboidal cells that are of pivotal importance for photoreceptor health (Strauss, 2005; Bazan et al., 2011). Firstly, this collaboration involves the initial DHA retention from the choroidal blood flow by the RPE and delivery to the photoreceptor inner segments. Here, DHA is mainly incorporated into phospholipids and used for POS assembly (Bazan et al., 2011; Shindou et al., 2017). Other important constituents of the POS phospholipids are VLC-PUFAs (C > 30). Their synthesis is largely mediated by the enzyme called elongation of very long chain fatty acids-4 (ELOVL4), possibly starting from different PUFAs [e.g., DHA and its β-oxidation product eicosapentaenoic acid (EPA, C20:5*n*-3)] (Agbaga et al., 2010). Secondly, via the daily phagocytosis of shed POS, the RPE recycles the PUFAs, which are re-used for new POS synthesis (Bazan et al., 2011). Although much information has been gathered on retinal PUFA homeostasis, important questions remain unanswered. Given the importance of peroxisomal β-oxidation for PUFA metabolism, a role in retinal PUFA homeostasis has repeatedly been suggested (Agbaga et al., 2010; Rice et al., 2015; Reyes-Reveles et al., 2017), but this was never investigated. Recently, we revealed a differential distribution of proteins involved in peroxisomal β-oxidation between the retina and RPE (Das et al., 2019). Together with the finding that photoreceptor inner segments and the RPE are the cell types of the retina most enriched in peroxisomes (Smith et al., 2016; Zaki et al., 2016; Argyriou et al., 2019; Daniele et al., 2019; Das et al., 2019), we propose that peroxisomal β-oxidation plays an important, yet distinct function in these cell types.

To decipher the importance of peroxisomal β-oxidation for retinal integrity, we here characterized the retinal phenotype of our previously developed MFP2 knockout mouse model (denoted as *Mfp2^–/–^* mice) (Baes et al., 2000). Using *in vivo*, (immuno)histochemical and biochemical analyses, we revealed a complex retinal phenotype, consisting of a developmental defect in photoreceptor maturation and a progressive deterioration of the retina and RPE. Our observations prove that peroxisomal β-oxidation is crucial for retinal health in mice.

## Materials and Methods

### Mouse Breeding

In view of their impaired fertility, *Mfp2^–/–^* mice were generated from heterozygous breeding pairs and identified by genotyping, as described (Baes et al., 2000). Upon comparison of the retinal morphology, gene expression and lipid composition, we observed no differences between *Mfp2^+/+^* and *Mfp2^+/–^* mice, and, therefore, both genotypes were used as control for the *Mfp2^–/–^* mice. All mice were bred in a C57Bl6/J background. Animals were bred in the animal housing facility of the KU Leuven, had *ad libitum* access to water and standard rodent food and were kept on a 14/10 h light and dark cycle. All mice were sacrificed between 2 and 6 pm. Mice were anesthetized by an intraperitoneal injection of a mixture of medetomidine (1 mg/kg; Domitor^®^, Orion Pharma) and ketamine (75 mg/kg; Nimatek^®^, Dechra) and sacrificed via cervical dislocation, unless stated otherwise. All experiments were in accordance with the Association for Research in Vision and Ophthalmology (ARVO) Statement for the use of Animals in Ophthalmic and Visual Research, the Guidelines for Care and Use of Experimental Animals (NIH) and the European Directive 2010/63/EU, and were fully approved by the Research Ethical Committee of the KU Leuven (P166/2017).

### *In vivo* Tests

Visual acuity was assessed by measuring the optokinetic tracking response, which is a visually evoked head movement in the same direction as a stimulus when the latter is detected by the mouse. The optokinetic tracking response was measured in photopic conditions, as previously described (Prusky et al., 2004).

Retinal functionality was assessed via ERG, using the Celeris system (Diagnosys). Briefly, the mice were overnight dark-adapted and subsequently anesthetized. Body temperature was maintained at 37°C. Pupils were dilated with 0.5% tropicamide (Tropicol^®^, Thea Pharma) and 15% phenylephrine (Thea Pharma). Genteal drops (Novartis) were used to keep the eyes moist during the experiment and also served as conductor of the electrical signal between the eyes and the electrodes. To determine the scotopic ERG responses (rod-mediated), five recordings at three increasing flash intensities (0.01–1.0 cd^∗^s/m^2^) were measured and averaged per intensity. Subsequently, the eyes were subjected to light (9 cd^∗^s/m^2^ for 10 min) and photopic ERG responses (cone-mediated) were measured. Hereto, single flashes at 3 and 10 cd^∗^s/m^2^ were applied. Subsequently, a- and b-wave amplitudes were calculated by the software, representing, respectively, photoreceptor and interneuron responses. The latter mostly comprises the bipolar cell response, and reflects both photoreceptor-to-bipolar cell signal transmission and bipolar cell function (Kinoshita and Peachey, 2018). The a-wave amplitude was calculated from the baseline to the trough of the negative peak, whereas the b-wave was calculated from the trough of the negative peak to the crest of the positive peak. Finally, data from the two eyes were averaged and used in the analyses.

### Histopathology

Enucleated eyes were processed to generate transverse retinal paraffin sections (7 μm). Hereto, eyes were fixed overnight at 4°C in Modified Davidson’s Fixative (DF) [22.2% (v/v) formaldehyde 10%, 32% (v/v) ethanol, 11.1% (v/v) glacial acetic acid]. Gross morphology was assessed by standard hematoxylin-eosin (H&E) staining, followed by morphometric analyses in ImageJ (NIH). The number of photoreceptor nuclei were counted over a distance of 100 μm at 6 different regions: central [± 200 μm from optic nerve head (ONH)], middle (± 1000 μm from ONH) and peripheral region (± 100 μm from the edge of the retina) in both the nasal and temporal plane. Images were acquired with an inverted IX-81 microscope (Olympus, 20× objective: UPLFLN20×).

Photoreceptor layer (PR layer), POS and photoreceptor inner segment (PIS) length was assessed by phase-contrast microscopy. Paraffin sections were deparaffinized with xylene (100%), and rehydrated in decreasing concentrations of ethanol [100, 90, 70, and 50% (v/v)] and distilled water. Subsequently, the sections were mounted with ProLong^®^ Gold antifade mountant (Invitrogen). Images were acquired with a Leica DMI6000B microscope, using phase-contrast settings (63× objective: HCX PL Fluotar 63×/1.25 oil PH3). Per mouse, one image in the middle region of either plane (temporal and nasal) was obtained and layer thickness was measured in ImageJ (NIH). On each image three non-adjacent regions were measured. Finally, values from the two planes were averaged and used in the analysis.

Immunohistochemistry (IHC) on retinal paraffin sections was performed as previously described (Das et al., 2019), with minor modifications. Briefly, after deparaffinization and rehydration, antigen retrieval was performed by heating the sections in 1 mM EDTA solution (pH 8.0) in the microwave for 10 min. In case an HRP-conjugated secondary antibody was used, endogenous peroxidases were inactivated by incubating the sections for 30 min in 3% (v/v) H_2_O_2_. Subsequent to a blocking step with 2% (v/v) normal goat serum, the sections were overnight incubated at 4°C with the primary antibody. Next, an appropriate secondary antibody was applied: anti-rabbit or anti-mouse HRP conjugated IgG (1/200) (Agilent), AlexaFluor 488 goat anti-rabbit IgG or AlexaFluor 568 goat anti-mouse IgG (1/200) (Agilent). In case of an HRP-conjugated secondary antibody, the fluorescein TSA plus amplification kit (Perkin Elmer) was used, according to the manufacturer’s instructions. The primary antibodies and the dilutions are summarized in Table 1. To assess apoptotic cell death, terminal deoxynucleotidyl transferase dUTP nick end labeling (TUNEL) staining was performed on retinal paraffin sections, using the *in situ* cell death detection kit (Roche), according to manufacturer’s instructions. The sections were counterstained with Hoechst 33342 and mounted with ProLong^®^ Gold antifade mountant. Images were acquired with a Leica SP8x confocal microscope (40× objective: HC PL APO 40×/1.30 Oil CS2).

**TABLE 1:** List of antibodies used for IHC and Western blotting (WB).

Primary antibody	Host	Dilution IHC (secondary antibody)	Dilution WB	Supplier
ADIPOR1	Rabbit	−	1/100	Tecan (18993)
GFAP	Rabbit	1/10,000 (HRP)	−	Dako (Z0344)
Iba1	Rabbit	1/500 (HRP)	−	Wako (019-19741)
Peanut agglutinin (FITC-linked)	−	1/100	−	Vector Laboratories (FL-1071)
Recoverin	Rabbit	−	1/1,000	Millipore (AB5585)
Rhodopsin	Mouse	1/1,000 (Alexa, 1/750)	1/2,000	Millipore (MAB5356)
Vinculin	Mouse	−	1/2,000	Sigma (V9131)
β-actin	Mouse	−	1/5,000	Abcam (8226)

### Transmission Electron Microscopy

After deep anesthesia, the mice were transcardially perfused with Hanks’ balanced salt solution (HBSS, Gibco) supplemented with heparin (5 U/mL, Leo Pharma), followed by the fixative solution consisting of 2.5% (v/v) glutaraldehyde in 0.05 M sodium cacodylate (pH 7.3). Eyes were enucleated and dissected to remove the cornea and lens. Eyecups were further fixed overnight at 4°C, using the same fixative solution. Further processing of the eyecups was performed as previously described (Baboota et al., 2019). Transmission electron microscopy was performed on a JEOL JEM1400 (JEOL Europe BV) (VIB Bio Imaging Core, Leuven Platform).

### Western Blot Analyses

Western blotting was performed as previously described (Das et al., 2019). The primary antibodies and dilutions are summarized in Table 1. Importantly, samples were not boiled for the detection of Adiponectin receptor 1 (ADIPOR1), but kept at 37°C for 10 min, as previously described (Sluch et al., 2018). HRP-conjugated secondary antibodies (1/5,000, Agilent) were applied and immune-complexes detected with Amersham ECL Western Blotting Detection Reagent (GE Healthcare Life Science) using the ChemiDoc MP System (Bio-Rad). The images were processed with the Image Lab software (Bio-Rad). Vinculin and β-actin were used as loading control.

### RNA Analyses

Dissection for neural retina and RPE isolation was performed as previously described (Xin-Zhao Wang et al., 2012). Briefly, after removal of connective tissue, muscles, optic nerve and the anterior segment, neural retina was separated from the posterior eyecup (RPE/choroid/sclera), snap frozen in liquid nitrogen and stored at −80°C until use. Subsequently, the eyecup was dipped into PBS to remove any debris, transferred to 200 μL of ice-cold RNAprotect cell reagent (Qiagen) and incubated for 10 min with gentle agitation every 1–2 min to release the RPE cells. Next, the eyecup was removed and the RPE cells pelleted by centrifugation (5 min at 600 g). Finally, RNA was extracted from the pelleted RPE cells using the PureLink RNA Mini Kit (Thermo scientific), according to manufacturer’s instructions. In contrast, the snap-frozen neural retinas were first homogenized in Trizol (Thermo Fisher Scientific), with subsequent RNA extraction using the PureLink RNA Mini Kit. In addition, DNase treatment was incorporated during RNA isolation of samples used for RNA sequencing. RNA concentration was measured using the NanoDrop 1000 spectrophotometer (Thermo Fisher Scientific).

After the conversion of the RNA to cDNA using the QuantiTect reverse transcription Kit (Qiagen) according to the manufacturer’s instructions, Real-Time quantitative PCR (RT-qPCR) was performed using PowerUp SYBR Green Master Mix (Thermo Fisher Scientific) and the ABI PRISM 7500 Real Time PCR system (Applied Biosystems). Relative expression to a reference gene (*Actb*) was calculated using the 2^–ΔΔCT^-method. All primers were obtained from IDT and listed in Table 2.

**TABLE 2:** List of primers used for RT-qPCR.

	Primer sequences (5′–3′)	

Gene	Forward	Reverse
*Actb*	ATTGGCAACGAGCGGTT	AGGTCTTTACGGATGTCAACG
*Adipor1*	CTCATCTACCTCTCCATCGTCT	GTACAACACCACTCAAGCCA
*Mfsd2a*	GTCCCTATCATCCTCATCTTGC	GAGTCTGTATCCGAGCAACC

RNA sequencing experiments (executed by Genomics Core Leuven) were performed on RNA prepared from 3 weeks-old retinas. Per sample, 2 retinas of each mouse were pooled. The sequence libraries were prepared with the QuantSeq 3′ mRNA-Seq Library Prep Kit (Lexogen), according to the manufacturer’s protocol. Samples were indexed to allow for multiplexing. Library quality and size range were assessed with the DNA 1000 kit (Agilent Technologies), using a Bioanalyzer (Agilent Technologies). Subsequently, libraries were sequenced on a HiSeq4000 instrument (Illumina). Hereto, single-end reads of 50 base pairs were produced with a minimum of 1 million reads per sample. After quality control of the raw reads, alignment against the mouse reference genome and quantification of reads per gene, biostatistical analyses were performed using the R-based software package BIOMEX (Taverna et al., 2020). Here, differential expression analysis was performed using the Limma package and the reported *P*-values were adjusted for multiple testing with the Benjamini-Hochberg procedure to control the false discovery rate. Pathway enrichment was analyzed by Gene Set Enrichment Analysis (GSEA) against the Kyoto Encyclopedia for Genes and Genomes (KEGG) database.

*In situ* hybridization was performed using the RNAScope 2.5 HD Chromogenic Detection Kit (Advanced Cell Diagnostics), according to the manufacturer’s protocol. Hereto, enucleated eyes were fixed in 4% (w/v) paraformaldehyde in phosphate buffered saline for 18 h at 4°C, and embedded in paraffin. Six micrometer thick transverse retinal sections were hybridized with the *Mfp2* probe, designed by Advanced Cell Diagnostics, followed by amplification steps and chromogenic detection with Fast red. A positive [directed to cyclophilin B (PPIB)] and a negative [directed to Bacillus subtilis dihydrodipicolinate reductase (dapB)] control probe were included in each experiment. Images were acquired with a Leica SP8× confocal microscope (40× objective: HC PL APO 40×/1.30 Oil CS2).

### Lipid Analyses

For all lipid analyses, mice were sacrificed by cervical dislocation without anesthesia, to avoid a possible confounding effect of the anesthetics on the lipid composition. Neural retinas were homogenized in a medium containing 250 mM sucrose, 5 mM MOPS (pH 7.2), 1 mM EDTA, and 0.1% (v/v) ethanol. Blood was collected in heparinized tubes via a submandibular puncture and plasma was obtained by centrifugation (10 min at 1,000 g at 4°C).

Total fatty acids were measured as previously described (Medema et al., 2016). Briefly, after transmethylation in the presence of the internal standard, i.e., the methyl ester of 18-methylnonadecanoic acid, the homogenate was extracted with hexane. Eventually, the methylated fatty acids were separated by gas chromatography (GC) and detected by flame ionization detection (FID). By using the known amount of the internal standards, the fatty acids were calculated and subsequently normalized by the amount of protein. The double bond index (DBI) was calculated by taking the sum of the mole fractions of the different unsaturated fatty acids multiplied by their number of double bonds.

Plasma and tissue lipidomics were performed as previously described (Vaz et al., 2019). Briefly, the homogenates and plasma were subjected to a single-phase extraction with a chloroform-methanol mixture (1:1 v/v) in the presence of the appropriate internal standards. The lipid extracts were separated by both normal phase high-performance liquid chromatography (HPLC) and reversed phase ultra-performance liquid chromatography (UPLC), coupled to mass spectrometric detection in both positive and negative electrospray ionization mode. The datasets were processed using an in-house developed metabolomics pipeline written in the R programming language^1^. Of note, all values originating from this lipidomics approach were calculated relative to the proper internal standard. Summation of relative abundances of same class lipids to calculate total (phospho)lipid levels was performed with the assumptions of equal response to their respective internal standard and are by no means comparable between different species to compare relative concentrations. Only comparisons within the same species can be made between different sample groups (i.e., control vs. *Mfp2^–/–^*).

### Statistical Analyses

For each experiment, at least three animals were used, with a maximum of eight. The exact numbers are specified in the corresponding figure legends. Shapiro-Wilk test and *F*-test were used to assess the normal distribution of the data and the equality of the variances, respectively. Based on the design of the experiment, statistical analyses were performed using either the unpaired two-sided Student’s *t*-test, Mann-Whitney *U*-test or two-way ANOVA with Bonferroni multiple comparisons test. Statistical tests were carried out using the GraphPad Prism software (version 8.1). Data are expressed as mean ± SEM and statistical significance was set at *P* < 0.05.

## Results

### *Mfp2* Is Expressed Throughout the Retina

**FIGURE 1:**
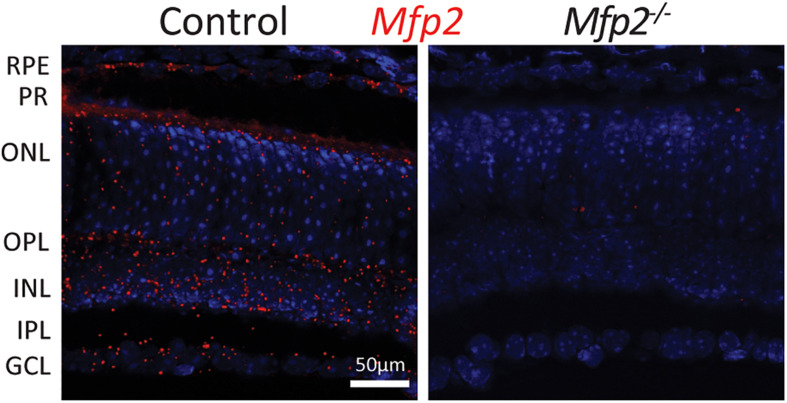
Spatial distribution of *Mfp2* transcripts in the mouse retina. Representative images of RNAscope^®^ *in situ* hybridization reveals extensive *Mfp2* expression throughout the retina of 9 weeks-old *Mfp2^+/+^* mice, but not in *Mfp2^–/–^* mice (*n* = 3 per genotype). Nuclei were counterstained with Hoechst (blue). RPE, retinal pigment epithelium; PR, photoreceptor layer; ONL, outer nuclear layer; OPL, outer plexiform layer; INL, inner nuclear layer; IPL, inner plexiform layer; GCL, ganglion cell layer.

We previously demonstrated by Western blot analysis that distinctive enzymes of the peroxisomal β-oxidation pathway are differentially distributed between the RPE and the neural retina, consisting of photoreceptors, interneurons and ganglion cells (Das et al., 2019). Due to the lack of suitable antibodies, immunohistochemical localization in the retinal layers could, however, not be performed. Therefore, we used the RNAscope^®^ *in situ* hybridization technique to detect the spatial distribution of *Mfp2* transcripts in the retina of 9 weeks-old wild type mice. We found abundant *Mfp2* expression in the RPE and in photoreceptor inner segments, but transcripts were also highly present in the outer and inner nuclear layer, outer plexiform layer and ganglion cell layer. Importantly, there was no specific labeling of *Mfp2* mRNA in the retina of mice in which MFP2 is inactivated (*Mfp2^–/–^* mice) (Figure 1).

### *Mfp2^–/–^* Mice Exhibit Decreased Retinal Function

**FIGURE 2:**
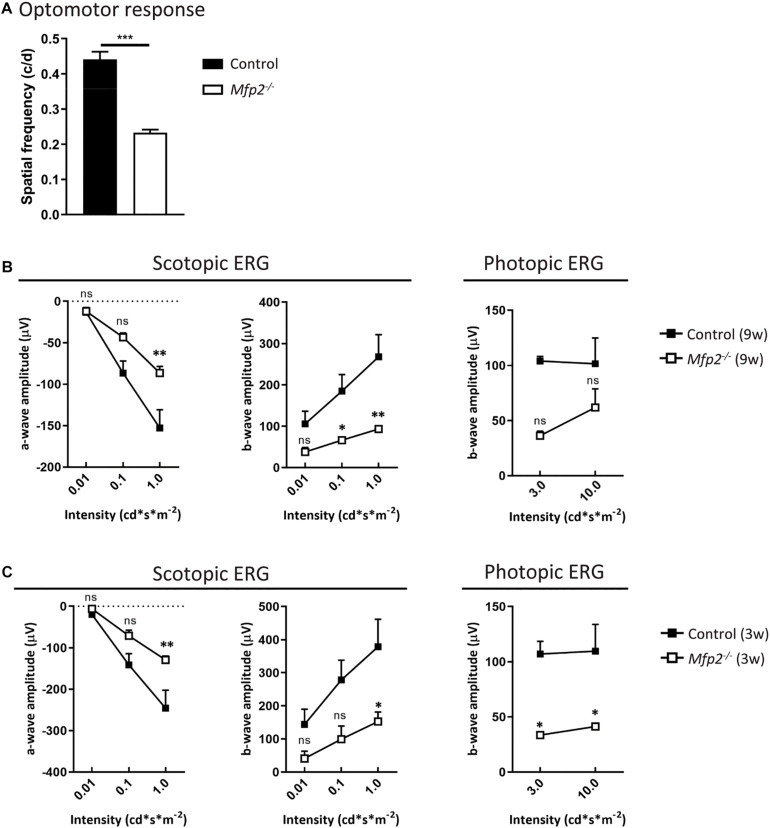
Decreased visual acuity and retinal function of *Mfp2^–/–^* mice. **(A)** Measurements of the optokinetic tracking response reveal reduced visual acuity in 9 weeks-old *Mfp2^–/–^* mice. ERG measurements show reduced scotopic a- and b-wave amplitudes and reduced photopic b-wave amplitudes in **(B)** 9 weeks-old, and **(C)** 3 weeks-old *Mfp2^–/–^* mice. Mean ± SEM are shown (*n* = 3–4 per genotype). Statistical significance was determined by two-way ANOVA with Bonferroni multiple comparisons test: ns *p* > 0.05, ^∗^*p* < 0.05, ^∗∗^*p* < 0.01, ^∗∗∗^*p* < 0.001.

To determine whether peroxisomal β-oxidation is essential for vision, we assessed the visual acuity of *Mfp2^–/–^* mice. The optokinetic tracking response was markedly (∼50%) reduced in 9 weeks-old *Mfp2^–/–^* mice compared to littermate controls (Figure 2A). To further examine whether this is related to dysfunction at the level of the retina, ERG experiments were conducted at the same age (Figure 2B). In scotopic (i.e., dark-adapted) conditions, the a-wave amplitude was significantly affected, indicative of impaired rod function. The b-wave, representing the inner retina response, was also reduced. In photopic (i.e., light-adapted) conditions, the decreased b-wave amplitudes indicated impaired cone function, although no statistically significant differences were reached. Upon testing at younger age, similar ERG abnormalities were observed in 6 weeks (data not shown) and 3 weeks-old *Mfp2^–/–^* mice (Figure 2C). Of note, at the younger ages, the changes in photopic b-wave did reach statistical significance. These data show that loss of MFP2 induces an early onset retinal dysfunction whereby the photoreceptors, interneurons and possibly Müller cells are affected.

### *Mfp2^–/–^* Retinas Display a Reduced Photoreceptor Length, Progressive Photoreceptor Degeneration and RPE Anomalies

**FIGURE 3:**
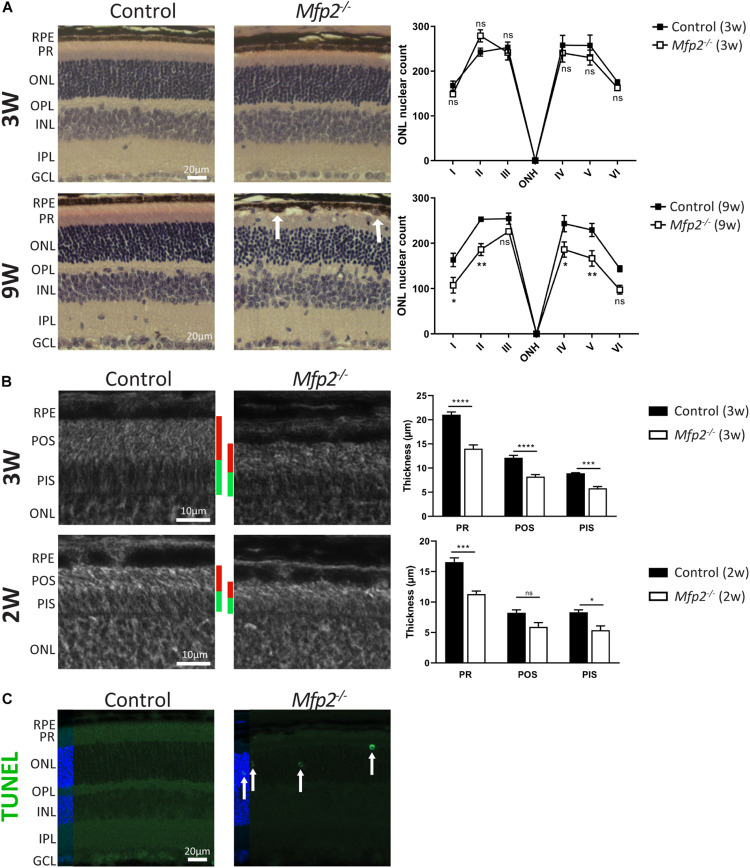
Morphological changes in *Mfp2^–/–^* retinas. **(A)** H&E staining reveals no gross morphological alterations, except for PR length reduction, in 3 weeks-old *Mfp2^–/–^* retinas and photoreceptor degeneration and protrusion of RPE cells in the POS layer (white arrows) in 9 weeks-old *Mfp2^–/–^* retinas. The spiderdiagrams show the quantification of the number of photoreceptor nuclei over a distance of 100 μm at six different positions: I, nasal-peripheral; II, nasal-middle; III, nasal-central; ONH, optic nerve head; IV, temporal-central; V, temporal-middle; VI, temporal-peripheral. **(B)** Phase-contrast microscopy of 3 and 2 weeks-old *Mfp2^–/–^* retinas show reduced photoreceptor outer (red bars) and inner segment (green bars) length. Mean ± SEM are shown (*n* = 3, 4 and 5 per genotype at 2, 3, and 9 weeks of age, respectively). Statistical significance was determined by two-way ANOVA with Bonferroni multiple comparisons test: ns *p* > 0.05, *p < 0.05, **p < 0.01, ***p < 0.001, ****p < 0.0001. **(C)** Increase in TUNEL-positive nuclei (white arrows) reveals an increase in apoptotic cell death of photoreceptors in 3 weeks-old *Mfp2^–/–^* mice (*n* = 3 per genotype). Hoechst (blue) was used as nuclear counter stain. RPE, retinal pigment epithelium; PR, photoreceptor layer; POS, photoreceptor outer segment; PIS, photoreceptor inner segment; ONL, outer nuclear layer; OPL, outer plexiform layer; INL, inner nuclear layer; IPL, inner plexiform layer; GCL, ganglion cell layer.

To examine which pathological alterations underlie the functional deficits, we conducted histological analyses by H&E staining and phase-contrast microscopy at the age of 3 and 9 weeks. The most obvious morphological abnormality in the retina of 3 weeks-old *Mfp2^–/–^* mice was a reduced thickness of the photoreceptor layer (Figures 3A,B). Morphological analysis on phase-contrast microscopy images revealed that the thickness of both outer and inner segments was reduced by ∼35% (Figure 3B). In contrast, the total retinal thickness was unchanged (data not shown) and the number and distribution of photoreceptor nuclei in the outer nuclear layer were normal compared to age-matched control mice (Figure 3A). To investigate whether the decrease in photoreceptor outer and inner segments was caused by a developmental deficiency or by a degenerative process after eye opening, we assessed retinal morphology of 2 weeks-old *Mfp2^–/–^* mice. Already at this age, the size of the photoreceptor layer was reduced by ∼30%, suggesting that it was caused by a developmental problem (Figure 3B).

Histological analysis at the age of 9 weeks revealed a marked deterioration of the neural retina (Figure 3A). The POS length reduction progressed variably including areas with severe shortening and areas where no further progression was observed. The inner segment length normalized compared to age-matched controls (data not shown). The distribution of nuclei in the outer nuclear layer was aberrant, and, upon counting, the number of nuclei was significantly reduced, pointing to progressive photoreceptor degeneration. By performing TUNEL staining at the age of 3 weeks, we proved that apoptotic death of photoreceptors was already going on at an earlier age (Figure 3C). In addition, at multiple locations spread over the entire retina, the RPE seemed to protrude into the POS layer (Figure 3A).

These results revealed that *Mfp2^–/–^* mice exhibit a complex retinal phenotype with a developmental defect in photoreceptor maturation and a progressive deterioration of photoreceptors and RPE cells. In the present study, we focused our analyses on the early onset abnormalities in the neural retina, while the progressive RPE phenotype will be the subject of future investigations.

### Transmission Electron Microscopy Reveals Normal POS Ultrastructure in Juvenile *Mfp2^–/–^* Mice

**FIGURE 4:**
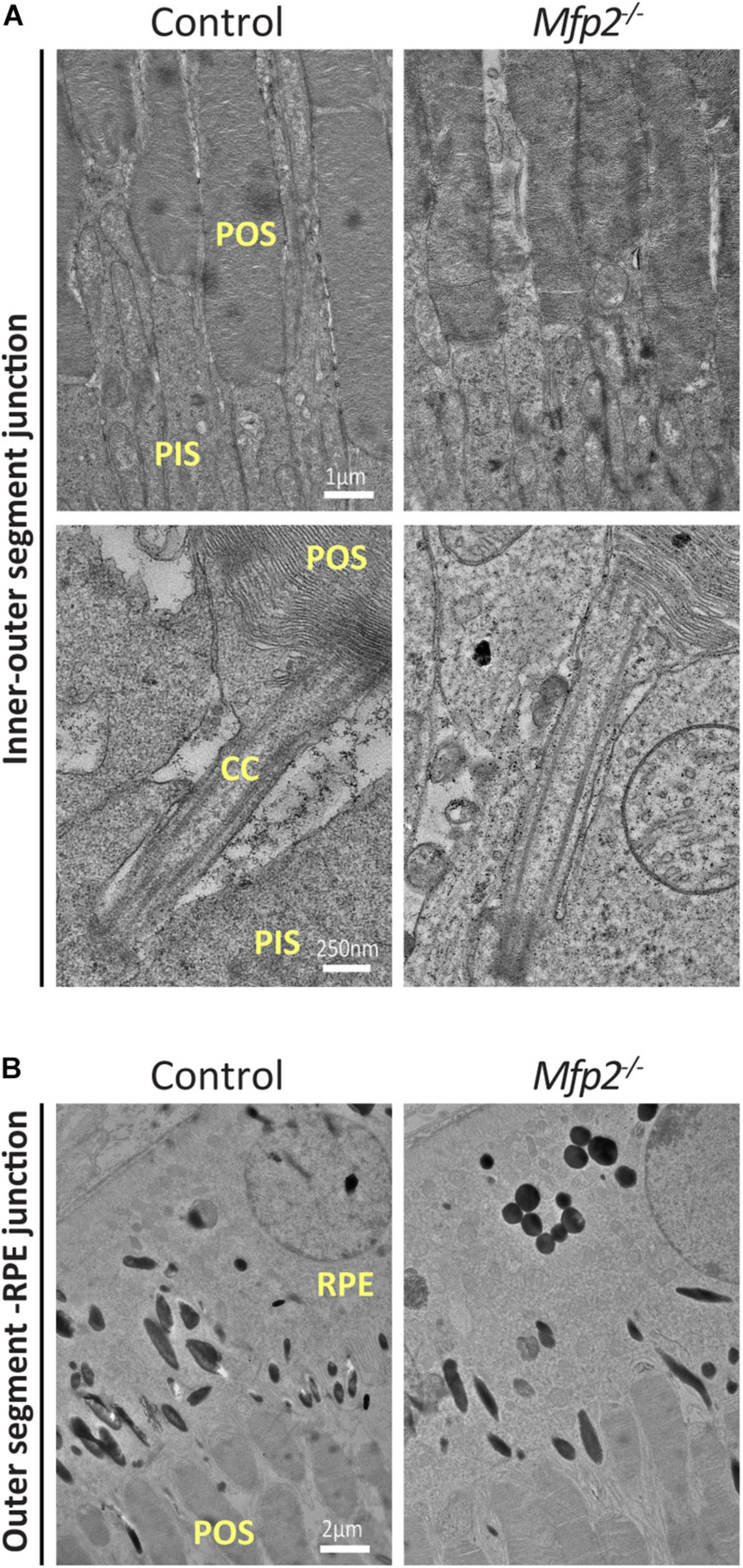
Normal POS formation in *Mfp2^–/–^* mice. TEM analysis reveals **(A)** unaltered POS disc organization and density and normal connecting cilium appearance and **(B)** normal contact of the POS with RPE cells in 3 weeks-old *Mfp2^–/–^* mice (*n* = 3-5 per genotype). CC, connecting cilium; POS, photoreceptor outer segment; PIS, photoreceptor inner segment; RPE, retinal pigment epithelium.

To better define the changes that take place in the photoreceptors of *Mfp2^–/–^* retinas, we performed transmission electron microscopy (TEM) analysis at the age of 3 weeks. Photoreceptor disc formation seemed to be unaffected, with normal connecting cilium (CC) appearance, disc organization and density. In addition, POS seemed to be properly connected with RPE cells (Figure 4). Mitochondria that are located in the inner segments showed normal morphology. In conclusion, TEM analysis suggests that, despite their shorter length, outer segments were normally formed.

### *Mfp2^–/–^* Mice Display a Change in Transcriptomic Profile in the Neural Retina

**FIGURE 5:**
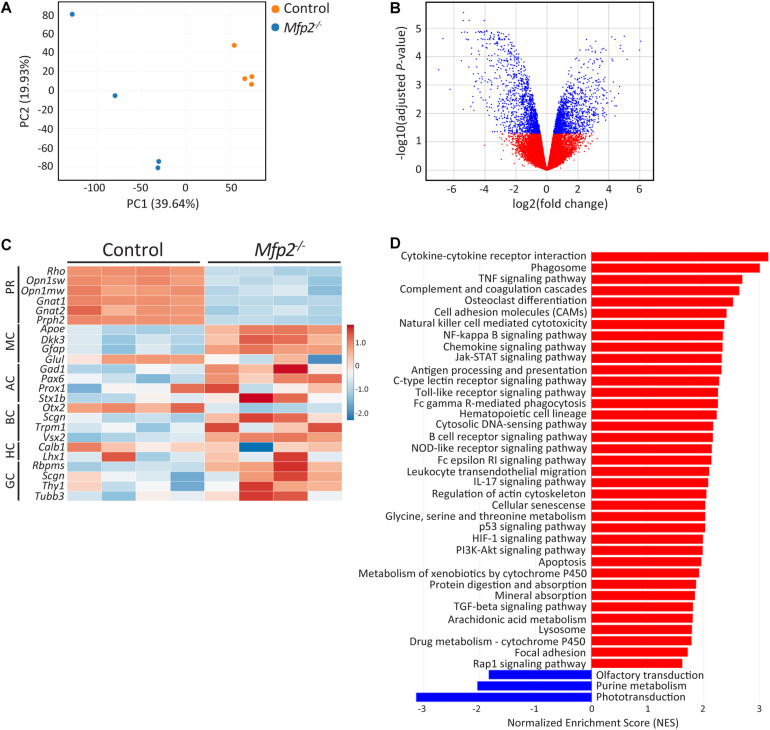
Distinct transcriptomic profile of *Mfp2^–/–^* retinas. **(A)** Principal component analysis (PCA) and **(B)** volcano plot of the differential expression analysis reveal a different transcriptomic signature of 3 weeks-old *Mfp2^–/–^* retinas. Blue and red dots in **(B)** represent differentially expressed and unchanged genes, respectively. **(C)** Heat map showing the expression of selected cell type specific genes. **(D)** GSEA reveals a major downregulation in the phototransduction pathway and a major upregulation in cell death and inflammation related pathways. Blue and red bars represent down- and upregulated pathways, respectively (*n* = 4 per genotype) PR, photoreceptors; MC, Müller cells; AC, amacrine cells; BC, bipolar cells; HC, horizontal cells; GC, ganglion cells.

The early onset retinal structural and functional deficits were further investigated by performing transcriptomic analysis of neural retinas at the age of 3 weeks. Principal component analysis revealed that *Mfp2^–/–^* retinas had a distinct transcriptomic signature compared to control retinas (Figure 5A). This observation was confirmed by the differential gene expression analysis, showing in total 2,894 differentially expressed genes (adjusted *P* < 0.05; 1468 genes upregulated and 1,426 genes downregulated) (Figure 5B). Next, we checked the expression of a subset of marker genes of the major retinal cell types (Figure 5C). Genes supporting photoreceptor morphology (e.g., *Prph2*) and function (e.g., *Rho, Opn1sw*, and *Opn1mw*) were severely downregulated in the mutant retinas. In addition, gene expression of other retinal cell types, i.e., bipolar (e.g., *Otx2* and *Vsx2* down and upregulation, respectively), amacrine (e.g., *Pax6* upregulation) and ganglion cell (e.g., *Tubb3* and *Thy1* upregulation), was also altered by MFP2 deletion. This suggests that these cells are also affected, which may relate to the impaired b-wave in the ERG. Subsequently, gene set enrichment analysis (GSEA) using the KEGG database was performed to detect dysregulated pathways (Figure 5D). The most important downregulated pathway was *phototransduction*. Other downregulated pathways, *olfactory transduction* and *purine metabolism*, represented genes that overlapped with *phototransduction* and pointed to impaired cGMP signaling, which is essential in phototransduction. In addition, GSEA revealed that most of the upregulated pathways were involved in inflammation related processes and in cell death (e.g., *apoptosis* and *p53 signaling* pathway). The strong upregulation of the phagosome pathway most likely relates to the upregulation of inflammatory pathways.

**FIGURE 6:**
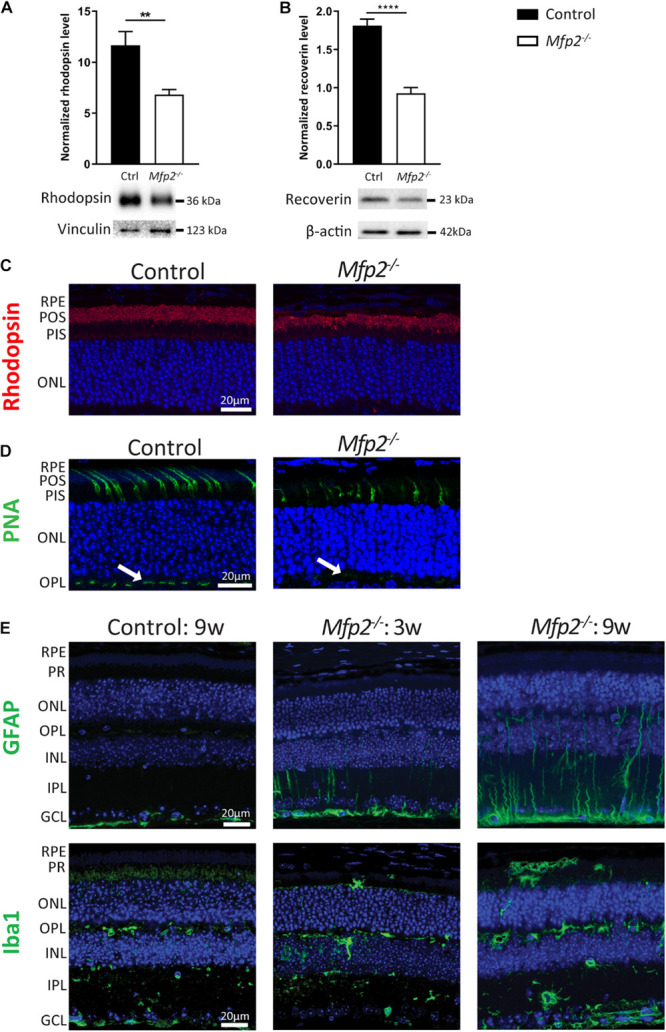
MFP2 ablation affects both rods and cones and causes secondary glial activation. Western blot analysis of **(A)** rhodopsin and **(B)** recoverin reveal decreased protein levels in 3 weeks-old *Mfp2^–/–^* retinas. Vinculin and β-actin are used as loading control in **(A,B)**, respectively. Mean ± SEM are shown (*n* = 5-9 per genotype). Statistical significance was determined by unpaired *t*-test: ** *p* < 0.01, **** *p* < 0.0001. **(C)** IHC for rhodopsin reveals shorter POS length and no mislocalization of the protein. **(D)** PNA-labeled cones exhibit a reduction in length in *Mfp2^–/–^* retinas. Cone pedicles in the OPL (white arrows) show a decrease in signal intensity. **(E)** IHC for GFAP and Iba1 shows the activation of Müller cells and microglia, respectively, in the MFP2 deficient retinas of 3 and 9 weeks-old mice. In addition to their activation, microglia also migrate toward the subretinal space. Hoechst (blue) was used as nuclear counterstain (*n* = 3 per genotype). RPE, retinal pigment epithelium; POS, photoreceptor outer segments; PIS, photoreceptor inner segments; ONL, outer nuclear layer; OPL, outer plexiform layer; INL, inner nuclear layer; IPL, inner plexiform layer; GCL, ganglion cell layer.

Given the extensive downregulation of genes encoding proteins that play a crucial role in phototransduction, Western blot analysis was performed at the age of 3 weeks, confirming that levels of the phototransduction proteins rhodopsin (rods) and recoverin (rods and cones) were reduced by ∼40 and ∼50%, respectively (Figures 6A,B). In addition, we performed IHC for rhodopsin (Figure 6C). Interestingly, quantification of the signal intensity per unit area in the POS showed no difference between *Mfp2^–/–^* and control retinas (data not shown), suggesting that the decrease in rhodopsin protein levels reflected the POS length reduction. Moreover, no mislocalization of rhodopsin was observed, suggesting normal connecting cilium function, which is in line with the normal ultrastructure. Furthermore, we labeled cones with FITC-linked peanut agglutinin (PNA) that binds to the extracellular matrix of cone outer segments and pedicles (Figure 6D). Similar to the rods, the cones were reduced in length in the *Mfp2^–/–^* retinas. Interestingly, the cone pedicles, located in the outer plexiform layer, exhibited a decreased signal intensity, which suggests that cone synapses are affected. The coordinate suppression of genes involved in phototransduction raised the question whether dedicated transcription factors (Swaroop et al., 2010) were also repressed. Indeed, transcripts of *Crx*, *Nrl*, *Nr2e3*, *Rorb*, and *Thrb* were all strongly reduced (data not shown).

The upregulation of cell death related processes was in line with the progressive photoreceptor degeneration (Figure 3A) and the increase in TUNEL-positive photoreceptor nuclei (Figure 3C). To elaborate on these observations, we performed IHC for the retinal stress marker glial fibrillary acidic protein (GFAP) (Figure 6E). More specifically, Müller cells, a type of retinal glial cells, become GFAP positive upon various triggers of retinal stress (Bramall et al., 2010). At the age of 3 weeks, GFAP-reactive Müller cells were detected, and this became more prominent in 9 weeks-old *Mfp2^–/–^* retinas. To validate the major increase in inflammation related pathways, we performed IHC for ionized calcium binding adaptor molecule 1 (Iba1), an established microglial marker. In the *Mfp2^–/–^* retina, reactive microglia with hypertrophic cell bodies and thickened processes were observed. Moreover, microglia were found to migrate toward the subretinal space, which is the space between the POS and the RPE. These observations were even more pronounced in the 9 weeks-old *Mfp2^–/–^* retinas (Figure 6E).

These data show that already at the age of 3 weeks *Mfp2^–/–^* neural retinas display important adaptations in gene expression. This involves several retinal cell types but alterations in photoreceptor gene expression were most prominent. In addition, loss of MFP2 elicited activation of inflammatory processes in the retina.

### *Mfp2*^–/–^ Retinas Exhibit an Altered PUFA Profile

To further search for mechanisms explaining the early onset retinal abnormalities, and in view of the role of peroxisomal β-oxidation in lipid homeostasis, we performed in-depth lipid analyses on neural retinas of 3 weeks-old *Mfp2^–/–^* mice.

**FIGURE 7:**
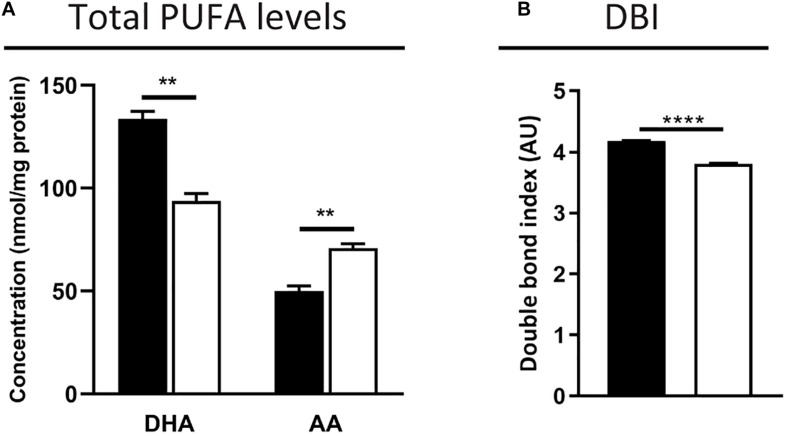
DHA decrease is partially compensated by an increase of *n*-6 PUFAs in *Mfp2^–/–^* retinas. **(A)** Total fatty acid measurements reveal a ∼30% decrease in DHA and a compensatory increase in *n*-6 PUFAs, such as AA, in 3 weeks-old *Mfp2^–/–^* retinas. **(B)** Calculation of the double bond index (DBI) shows a 10% decrease in *Mfp2^–/–^* retinas. Mean ± SEM are shown (*n* = 3 per genotype). Statistical significance was determined by unpaired *t*-test: ** *p* < 0.01, **** *p* < 0.0001.

First, we measured the total fatty acid concentration in the neural retina by GC. As expected, levels of the peroxisomal β-oxidation substrate C24:0 were elevated (2.5-fold increase vs. control mice, 0.8 ± 0.1 vs. 0.3 ± 0.0 nmol/mg tissue, *p* < 0.001). The POS are known to contain high PUFA levels, including DHA (C22:6*n*-3) amounting to approximately 50% of the POS phospholipid fatty acid side chains, and VLC-PUFAs (C > 30), which are low abundant *n*-3 and *n*-6 elongation products (Anderson and Maude, 1972; Tinoco, 1982; Fliesler and Anderson, 1983). The total DHA content in the *Mfp2^–/–^* retinas was decreased by ∼30%, which was accompanied by an increase of *n*-6 fatty acids, such as arachidonic acid (AA, C20:4*n*-6, ∼40% increase) (Figure 7A). The latter probably reflects a compensatory mechanism to maintain the retinal desaturation level. Indeed, calculation of the double bond index revealed a decrease of only 10% in the *Mfp2^–/–^* retinas, suggesting that the reduction in DHA was mostly compensated (Figure 7B). Unfortunately, VLC-PUFAs were not analyzed in the GC analysis.

**FIGURE 8:**
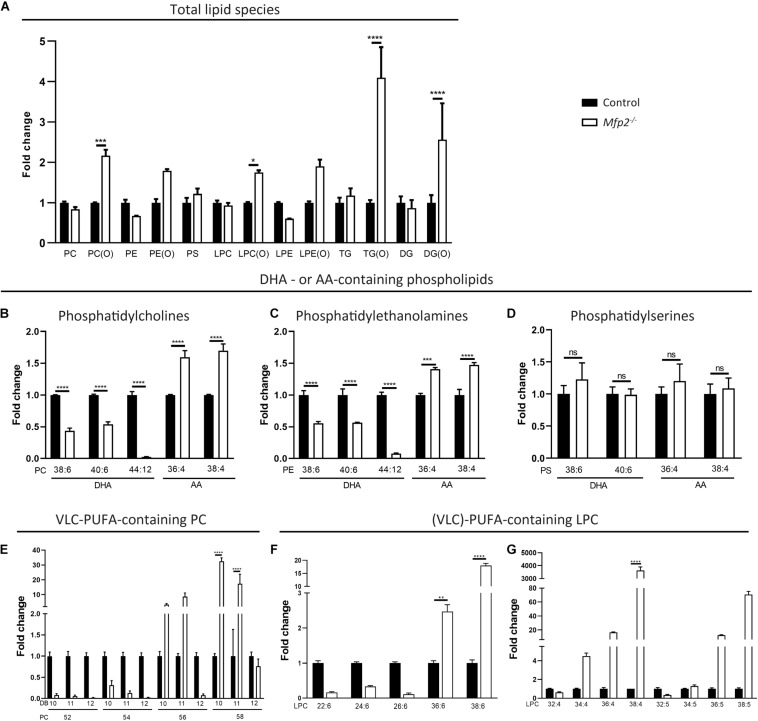
Altered PUFA profile in 3 weeks-old *Mfp2^–/–^* retinas. **(A)** Evaluation of the different lipid classes reveals a general increase in ether lipid species. Lipidome analysis reveals a severe decrease in DHA- and an increase in AA-containing **(B)** PC and **(C)** PE phospholipids, but not in **(D)** PS phospholipids. **(E)** *Mfp2^–/–^* retinas display a peculiar profile of PC phospholipids containing VLC-PUFAs. PC species with 52–58 carbons and 10–12 double bonds (DB) are shown. Profile of LPC species containing PUFAs with **(F)** 6 and **(G)** 4–5 double bonds. Mean ± SEM are shown (*n* = 3 per genotype). Statistical significance was determined by two-way ANOVA with Bonferroni multiple comparisons test: ns *p* > 0.05, **p < 0.01, ***p < 0.001, ****p < 0.0001. PC, phosphatidylcholines; PE, phosphatidylethanolamines; PS, phosphatidylserines; TG, triglycerides; DG, diacylglycerides; LPC/LPE, lyso-variant of PC/PE; x(O), ether-variant of the respective lipid species.

Subsequently, a comprehensive lipidomic analysis of the neural retina was performed. To obtain an overview of relative changes in the different lipid classes, the sum of the calculated signals of the different subspecies per group were compared. No large differences were observed, except a general increase in ether lipid species. More specifically, the ether variants of (lyso)PC and of di- and triglycerides were ∼2–5-fold increased (Figure 8A).

Given the importance of PUFAs in POS phospholipids and the potential role of peroxisomal β-oxidation in their homeostasis, our further analysis focuses on these PUFA-containing phospholipid species, although miscellaneous changes in other fatty acid species were detected (data not shown). While the lipidomic profiling does not allow to unequivocally identify DHA-containing phospholipids, PC species with 6 double bonds and a total carbon length of 38 or 40 likely contain one DHA in combination with a common saturated fatty acid with 16 or 18 carbons, respectively. Similarly, AA-containing phospholipids can be identified. In line with the GC data, PC(38:6) and PC(40:6) were reduced ∼2-fold, whereas PC(36:4) and PC(38:4) were increased ∼1.5-fold. More striking was the virtual depletion of PC(44:12), compatible with two esterified DHA moieties (Figure 8B). Similar changes were observed in the corresponding PE phospholipids, but not in the PS species (Figures 8C,D). Furthermore, PC species probably composed of DHA and a VLC-PUFA elongated from DHA up to a length of 34 carbons [e.g., PC(52:12), PC(54:12), and PC(56:12)] were almost absent in the *Mfp2^–/–^* neural retina, while those containing even longer VLC-PUFAs (≥C36) were unaltered or even increased. In addition, PC species with 10 double bonds (most likely DHA + VLC-PUFA elongated from AA) and 11 double bonds (most likely DHA + VLC-PUFA elongated from C20:5*n*-3 or C22:5*n*-6) displayed a similar trend as the PC species with 12 double bonds (Figure 8E). On the other hand, PC species with 4–7 double bonds were increased (data not shown), probably to compensate for the decreased abundance of the DHA-containing phospholipids and to maintain the retinal desaturation level.

Inspection of the lyso-variant of the phospholipids allows unambiguous identification of the esterified fatty acid. First, in line with the defect in peroxisomal β-oxidation, the disease marker, LPC(26:0) (Jaspers et al., 2020), was ∼150-fold (± 40-fold, *p* < 0.001) increased in the *Mfp2^–/–^* neural retina. LPC(22:6) and the LPC species containing the DHA-elongation products C24:6 and C26:6 [LPC(24:6) and LPC(26:6)] were reduced, whereas LPC(36:6) and LPC(38:6) were increased, confirming the changes in the PC species (i.e., increase starting from C36) (Figure 8F). Furthermore, VLC-PUFAs with 4 and 5 double bonds showed an increase starting from 34 carbons, also confirming the changes observed in the PC species (Figure 8G). The marked accumulation of PUFAs with ultra-long chains (≥ C34) raised the question whether this was related to increased expression of the *Elovl4* gene in the retina, given its role in the elongation of fatty acids containing ≥ 26 carbons (Agbaga et al., 2010). Interestingly, according to the RNAseq data, *Elovl4* transcripts were ∼4-fold decreased (data not shown).

Overall, the lipid analyses reveal that 3 weeks-old *Mfp2^–/–^* mice display important changes in the retinal lipid composition, with pronounced reductions in DHA-containing phospholipids and a peculiar VLC-PUFA profile.

### Retinal DHA Supply and Traffic Are Hampered in *Mfp2*^–/–^ Mice

**FIGURE 9:**
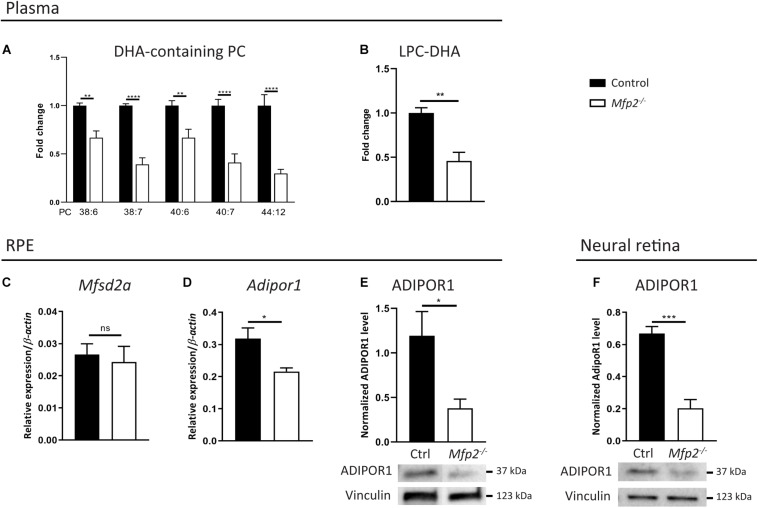
Systemic delivery and retinal traffic of DHA is impaired in *Mfp2^–/–^* retinas. Lipidome analysis reveals a decrease of DHA-containing **(A)** PC species and **(B)** LPC in plasma of 3 weeks-old *Mfp2^–/–^* mice. **(C,D)** Gene expression analysis by RT-qPCR reveals unchanged *Mfsd2a* and downregulated *Adipor1* expression in the RPE of *Mfp2^–/–^* mice. Western blot analysis for ADIPOR1 shows a 70% reduction in **(E)** the RPE and in **(F)** the retina of *Mfp2^–/–^* mice. Vinculin was used as loading control. Mean ± SEM are shown (*n* = 4–8 per genotype). Statistical significance was determined by unpaired *t*-test, Mann-Whitney *U*-test and two-way ANOVA with Bonferroni multiple comparisons test: ns *p* > 0.05, *p > 0.05, **p < 0.01, ***p < 0.001, ****p < 0.0001.

The virtual depletion of phospholipid species likely containing 1 or 2 DHA moieties in the neural retina of *Mfp2^–/–^* mice raised the question whether the supply and/or the uptake of DHA in the retina was impaired. Indeed, it is generally accepted that systemic supply accounts for the majority of the retinal DHA levels, over local synthesis (Bazan et al., 1982, 2011; Wang and Anderson, 1993; Rotstein et al., 1996; Simon et al., 2016). In plasma, DHA mainly resides in the phospholipid pool (Bazinet et al., 2019). Therefore, we first performed a lipidomic analysis on plasma of 3 weeks-old mice and found a ∼1.5–3-fold decrease in DHA-containing PC species (Figure 9A). This reduction was also observed in DHA incorporated in other common phospholipid classes (data not shown). In addition, we examined LPC(22:6), which is an important source for uptake of DHA from the blood into the eye (Wong et al., 2016; Lobanova et al., 2019). Also LPC(22:6) showed a ∼2-fold decrease in plasma (Figure 9B). These data suggest that systemic DHA-delivery to the eye was hampered.

Furthermore, several proteins dedicated to retinal DHA uptake and subsequent transport to the photoreceptors, more specifically Major facilitator superfamily domain-containing protein 2a (Mfsd2a) in the RPE (Wong et al., 2016; Lobanova et al., 2019) and ADIPOR1 in the RPE and in photoreceptors (Rice et al., 2015; Sluch et al., 2018), were recently identified. By RT-qPCR, we found that the gene expression of *Mfsd2a* and *Adipor1* in the RPE were, respectively, unchanged and reduced by ∼30% (Figures 9C,D). We validated the latter on the protein level and found a ∼70% reduction in ADIPOR1 levels (Figure 9E). The transcriptomic profiling of neural retinas revealed a ∼5-fold decrease of *Adipor1*, which was confirmed by a ∼70% reduction in ADIPOR1 protein levels (Figure 9F). Together, these results suggest that, in addition to a reduced systemic DHA supply, retinal traffic of this PUFA was likely impaired.

## Discussion

In this study, we have shown that intact peroxisomal β-oxidation is of crucial importance for retinal integrity. Mice lacking the pivotal peroxisomal β-oxidation enzyme MFP2 (*Mfp2^–/–^* mice) displayed a complex retinal phenotype. In juvenile mice, the proper formation of photoreceptors was impaired with reduced outer and inner segment length coinciding with profound lipid perturbations and altered gene expression. The transcriptome changes also indicated that other cell types of the inner retina were also affected. Furthermore, the *Mfp2*^–/^*^–^* retinas deteriorated upon aging, with progressive photoreceptor degeneration and RPE abnormalities. Here, we focused our analyses on the early onset photoreceptor anomalies.

At the age of 3 weeks, when retinal formation is completed in mice (Swaroop et al., 2010), the reduced size of inner and outer segment length in *Mfp2^–/–^* retinas pointed to a developmental problem. Yet, the retina was normally structured with unaltered numbers of photoreceptor nuclei and a normal ultrastructural appearance of photoreceptors, with intact connecting cilium and disc formation. These observations point to normal photoreceptor differentiation, but a hampered maturation. Despite the moderate structural defects, specifically in the inner and outer segments, photoreceptors exhibited marked transcriptome changes, affecting both rods and cones, and with severe downregulation of genes encoding phototransduction proteins. The latter was confirmed by a ∼40 and ∼50% reduction of rhodopsin and recoverin, respectively. Interestingly, rhodopsin is a major determinant of POS formation, which is underscored by their absence in mice homozygous for *rhodopsin* gene deletion (*Rho*^–/–^ mice) (Humphries et al., 1997; Lem et al., 1999). Moreover, it was shown that the length of the POS is directly proportional with increasing rhodopsin levels. Notably, juvenile *Rho*^+/–^ mice display a similar retinal phenotype compared to the *Mfp2^–/–^* mice, i.e., POS length shortening (Price et al., 2012). Besides the observed reduction in disc number, the general decrease in phototransduction proteins probably also contributed to the impaired ERG responses. This is supported by previous observations that *Rho*^+/–^ mice exhibited reduced electrophysiological responses (Liang et al., 2004). The important upregulation of genes involved in cell death related processes was confirmed by TUNEL staining and pointed to apoptotic photoreceptor death. This resulted in a disorganized outer nuclear layer containing fewer photoreceptor nuclei at the age of 9 weeks, which further worsened by the age of 16 weeks (data not shown). We did not thoroughly assess the retinal pathology at this later age, because the *Mfp2^–/–^* mice develop cataracts from around 11 weeks, which may impact on the retina. Moreover, later ages could not be investigated due to their early death at the age of 4–6 months, caused by various CNS defects (Baes et al., 2000; Huyghe et al., 2006).

A comprehensive lipidome analysis of the neural retina at the age of 3 weeks revealed a major shortage of glycerophospholipids likely containing one DHA and even depletion of those containing two DHA moieties. Although it may be argued that this is a consequence of the shortening of the outer segments, which are known to harbor these PUFA-containing phospholipids, this is presumably not the case because (i) the deficit of PC(44:12) is much more pronounced than the loss of outer segments and (ii) PC containing other PUFA species increased in content. Despite some recent new insights, the acquisition and homeostasis of DHA in the retina is still not fully understood. Besides some limited contribution by local synthesis, DHA is primarily taken up from the blood by the RPE through recently identified receptors, ADIPOR1 and Mfsd2a, and further shuttled to the photoreceptors (Bazan et al., 1982, 2011; Wang and Anderson, 1993; Rotstein et al., 1996; Rice et al., 2015; Simon et al., 2016; Wong et al., 2016; Lobanova et al., 2019). We found that DHA-containing phospholipids were reduced in the plasma of 3 weeks-old *Mfp2^–/–^* mice, suggesting a hampered systemic supply. This could result from impaired hepatic DHA synthesis, which depends on peroxisomal β-oxidation, but also from decreased intestinal absorption during the lactation period, as a result of disrupted bile acid synthesis and steatorrhea (Baes et al., 2000; Van Veldhoven, 2010). In addition, a marked reduction of ADIPOR1 in the RPE and neural retina suggested hampered retinal DHA trafficking (Rice et al., 2015).

POS also contain relatively high amounts of VLC-PUFAs, which are exclusively synthesized in the photoreceptor inner segments. DHA serves as the substrate for the elongation process, which is mediated by ELOVL4. These VLC-PUFAs are usually esterified to the *sn*-1 position of the phospholipids, with DHA occupying the *sn*-2 position (Agbaga et al., 2010). In the *Mfp2^–/–^* retinas, we observed a peculiar profile of those VLC-PUFA- and DHA-containing phospholipid species. More specifically, we found severe decreases in PC species containing up to 56 carbons, while those containing even more carbons were unchanged or elevated. Although it is difficult to pinpoint the underlying mechanism at this time, this may relate on one hand to the reduced levels of DHA, and on the other hand to the lack of peroxisomal β-oxidation, enabling an uncontrolled elongation of PUFA species. Unfortunately, our lipidome analysis has several limitations. Firstly, due to the difference in response factor of the different fatty acids in the MS analysis, the absolute concentrations of the numerous species cannot be retrieved. Moreover, the reported values are relative to the internal standard and data are presented as fold change compared to control. Secondly, since we only receive information about the total number of carbons and double bonds, we cannot unambiguously specify which PUFAs are exactly esterified in the phospholipids.

It is important to mention that DHA and VLC-PUFAs are converted by the RPE and photoreceptors into the lipid mediators, neuroprotectin D1 (NPD1) and elovanoids, respectively. Upon various degrees of retinal stress, these molecules exert anti-inflammatory and cytoprotective effects on both photoreceptors and RPE cells (Mukherjee et al., 2004; Kanan et al., 2015; Jun et al., 2017). Lack of DHA, altered VLC-PUFA levels and RPE/photoreceptor malfunction could lead to a deficit in the production of these lipid mediators, possibly contributing to the observed phenotype. Besides the deregulated homeostasis of PUFA species, another lipid perturbation was the vast accumulation of phospholipids containing saturated VLCFAs, such as C26:0. This could be deduced from the LPC(26:0) accumulation and is an expected consequence of peroxisomal β-oxidation dysfunction (Jaspers et al., 2020). An additional unexpected lipid anomaly is the important increase of several ether phospholipid classes. Two crucial enzymes of the ether lipid synthesis pathway reside in peroxisomes (Wanders and Waterham, 2006), but how failure of the β-oxidation pathway results in increased levels of ether lipids is currently obscure.

Taking all these lipidome changes in *Mfp2^–/–^* retinas at early age into consideration, we postulate that the altered lipid composition of the neural retina, which likely mostly pertains to photoreceptors, undermines photoreceptor function, structure and survival. However, it is still elusive which changes are pathogenic and which relate to a defect in the photoreceptors. The generation and analysis of mice with photoreceptor selective deletion of MFP2 should shed light on the role of peroxisomal β-oxidation in these cells.

Importantly, it was hypothesized already decades ago that retinal DHA deficiency plays an important role in the pathogenesis of the retinopathy in PBD and MFP2 patients. This is underscored by the frequent observations of reduced plasma DHA levels in both PBD and peroxisomal β-oxidation deficiency patients. Moreover, retinal DHA deficiency was found in a Zellweger syndrome patient (Martinez, 1992; Noguer and Martinez, 2010). However, caution is warranted. Firstly, it was shown that several MFP2 deficient patients with severe retinopathy had normal plasma DHA levels. However, PC species containing DHA, which is a more sensitive marker, were not determined. In addition, no information was available on their retinal DHA status (Ferdinandusse et al., 2006). Secondly, DHA supplementation to prevent deterioration of visual acuity in Zellweger spectrum disorder patients produced contradictory results. Although a stabilizing effect on the deteriorating retinal function in patients with a mild phenotype was first observed, a randomized, double-blind and placebo-controlled clinical trial detected no effect of DHA treatment on retinal function (Noguer and Martinez, 2010; Paker et al., 2010).

Finally, the question arises whether *Mfp2*^–/^*^–^* mice mimic the pathological events in patients with this gene defect. The retinopathy hallmarks consist of abnormal retinal pigmentation upon fundoscopic evaluation, with or without decreased ERG responses and visual acuity, and can also occur in milder cases (Ferdinandusse et al., 2006; McMillan et al., 2012; Lines et al., 2014; Amor et al., 2016). However, to date, there is a paucity of information on the histological changes in the retina of patients with MFP2 deficiency. For Zellweger syndrome, which is the most severe clinical presentation of PBD, a few old reports describe the histopathology of the retina. Here, RPE atrophy, bi-leaflet inclusions in the RPE, photoreceptor outer and inner segment degeneration and photoreceptor loss have been described (Cohen et al., 1983; Glasgow et al., 1987). More recently, interval spectral-domain optical coherence tomography also detected hyperreflective spots on the RPE protruding into the POS layer and outer retinal atrophy (Courtney and Pennesi, 2013). Ganglion cell, nerve fiber layer and optic nerve atrophy and macrophage infiltration in the retina were also described (Cohen et al., 1983; Glasgow et al., 1987; Courtney and Pennesi, 2013). Clearly, several of these findings are recapitulated in *Mfp2^–/–^* mice. Although several mouse models for peroxisomal disorders have been developed over the years, until now the retinal phenotype of only one has been described. More specifically, Argyriou et al. have extensively characterized the *Pex1* knock-in mouse, mimicking the most common human *PEX1* mutation (G843D) (Argyriou et al., 2019). Although juvenile *Pex1* knock-in mice also exhibited decreased retinal function and visual acuity, the morphological alterations were less severe compared to the *Mfp2^–/–^* mice. More specifically, these mice displayed reduced photoreceptor outer and inner segment length at the age of 2 weeks, which was completely normalized at 3 weeks of age. Furthermore, while cones were significantly affected, as shown by IHC for cone arrestin, rods seemed to be preserved. A possible explanation for the milder phenotype is that this *Pex*1 mutation leads to a subfunctional protein, which might still partially exhibit its normal function in peroxisome biogenesis. While the *Pex1* knock-in mouse model might reflect a mild disease course, it seems that the retinal phenotype of the *Mfp2^–/–^* mice corresponds fairly well with the pathological abnormalities observed in severe disease states. However, more information on the retinal histopathology of MFP2 deficient patients is required in order to draw firm conclusions about the translatability of the model.

In conclusion, we have shown that peroxisomal β-oxidation is crucial for retinal integrity, both during its formation and maintenance. To decipher the importance of retinal versus systemic β-oxidation dysfunction further investigations with cell type selective MFP2 inactivation are required. Additional research will also be necessary to elucidate whether the lipid perturbations underlie the observed morphological abnormalities and changes in gene expression.

## Data Availability Statement

The datasets of the RNA sequencing experiment generated for this study have been deposited in NCBI’s Gene Expression Omnibus (Edgar et al., 2002) and are accessible through GEO Series accession number GSE163770 (https://www.ncbi.nlm.nih.gov/geo/query/acc.cgi?acc=GSE163770). Other datasets are available on request to the corresponding author.

## Ethics Statement

The animal study was reviewed and approved by the Animal Ethics Committee–KU Leuven (P166/2017).

## Author Contributions

YD: mouse breeding and manipulation, design and execution of the experiments and data analysis and interpretation, writing of drafts. RNA sequencing was executed by the Genomics Core Leuven and data were analyzed by YD. DS: participation in experimental work. DS and SK: scientific discussions. SV: transmission electron microscopy. FV, EW, AHCVK, BJ, KD, and NB: lipid and bioinformatic analyses. LM: scientific discussions and supervision of *in vivo* experiments. PV: scientific discussions and supervision interpretation lipid analyses. MB: concept and supervision of the study. All authors commented on and approved the submitted manuscript.

## Conflict of Interest

The authors declare that the research was conducted in the absence of any commercial or financial relationships that could be construed as a potential conflict of interest.

## Footnotes

1 http://www.r-project.org

## Abbreviations

AA: arachidonic acid
ADIPOR1: adiponectin receptor 1
DHA: docosahexaenoic acid
ELOVL4: elongation of very long chain fatty acids-4
ERG: electroretinography
GSEA: gene set enrichment analysis
(L)PC/PE/PS: (lyso)phosphatidylcholine-ethanolamine-serine
MFP2: multifunctional protein 2
Mfsd2a: Major facilitator superfamily domain-containing protein 2a
POS/PIS: photoreceptor outer/inner segments
PUFA: polyunsaturated fatty acid
RPE: retinal pigment epithelium
TUNEL: terminal deoxynucleotidyl transferase dUTP nick end labeling
VLCFA: very long chain fatty acid
VLC-PUFA: very long chain polyunsaturated fatty acid.

## References

[B1] Agbaga M. P. Mandal M. N. Anderson R. E. (2010). Retinal very long-chain PUFAs: new insights from studies on ELOVL4 protein. *J. Lipid Res.* 51 1624–1642. 10.1194/jlr.R005025 20299492 PMC2882734

[B2] Amor D. J. Marsh A. P. Storey E. Tankard R. Gillies G. Delatycki M. B. (2016). Heterozygous mutations in HSD17B4 cause juvenile peroxisomal D-bifunctional protein deficiency. *Neurol. Genet.* 2:e114. 10.1212/nxg.0000000000000114 27790638 PMC5070413

[B3] Anderson R. E. Maude M. B. (1972). Lipids of ocular tissues. 8. The effects of essential fatty acid deficiency on the phospholipids of the photoreceptor membranes of rat retina. *Arch. Biochem. Biophys.* 151 270–276. 10.1016/0003-9861(72)90497-3 5044519

[B4] Argyriou C. D’Agostino M. D. Braverman N. (2016). Peroxisome biogenesis disorders. *Transl. Sci. Rare Dis.* 1 111–144. 10.3233/trd-160003 29152457 PMC5678237

[B5] Argyriou C. Polosa A. Cecyre B. Hsieh M. Di Pietro E. Cui W. (2019). A longitudinal study of retinopathy in the PEX1-Gly844Asp mouse model for mild Zellweger spectrum disorder. *Exp. Eye Res.* 186:107713. 10.1016/j.exer.2019.107713 31254513

[B6] Baboota R. K. Shinde A. B. Lemaire K. Fransen M. Vinckier S. Van Veldhoven P. P. (2019). Functional peroxisomes are required for β-cell integrity in mice. *Mol. Metab.* 22 71–83. 10.1016/j.molmet.2019.02.001 30795913 PMC6437690

[B7] Baes M. Huyghe S. Carmeliet P. Declercq P. E. Collen D. Mannaerts G. P. (2000). Inactivation of the peroxisomal multifunctional protein-2 in mice impedes the degradation of not only 2-methyl-branched fatty acids and bile acid intermediates but also of very long chain fatty acids. *J. Biol. Chem.* 275 16329–16336. 10.1074/jbc.M001994200 10748062

[B8] Bazan H. E. Careaga M. M. Sprecher H. Bazan N. G. (1982). Chain elongation and desaturation of eicosapentaenoate to docosahexaenoate and phospholipid labeling in the rat retina in vivo. *Biochim. Biophys. Acta* 712 123–128. 10.1016/0005-2760(82)90093-5 6288109

[B9] Bazan N. G. Molina M. F. Gordon W. C. (2011). Docosahexaenoic acid signalolipidomics in nutrition: significance in aging, neuroinflammation, macular degeneration. Alzheimer’s, and other neurodegenerative diseases. *Annu. Rev. Nutr.* 31 321–351. 10.1146/annurev.nutr.012809.104635 21756134 PMC3406932

[B10] Bazinet R. P. Bernoud-Hubac N. Lagarde M. (2019). How the plasma lysophospholipid and unesterified fatty acid pools supply the brain with docosahexaenoic acid. *Prostaglandins Leukot. Essent. Fatty Acids* 142 1–3. 10.1016/j.plefa.2018.12.003 30773208

[B11] Bramall A. N. Wright A. F. Jacobson S. G. McInnes R. R. (2010). The genomic, biochemical, and cellular responses of the retina in inherited photoreceptor degenerations and prospects for the treatment of these disorders. *Annu. Rev. Neurosci.* 33 441–472. 10.1146/annurev-neuro-060909-153227 20572772

[B12] Cohen S. M. Brown F. R. III Martyn L. Moser H. W. Chen W. Kistenmacher M. (1983). Ocular histopathologic and biochemical studies of the cerebrohepatorenal syndrome (Zellweger’s syndrome) and its relationship to neonatal adrenoleukodystrophy. *Am. J. Ophthalmol.* 96 488–501. 10.1016/s0002-9394(14)77913-9 6624831

[B13] Courtney R. J. Pennesi M. E. (2013). Interval spectral-domain optical coherence tomography and electrophysiology findings in neonatal adrenoleukodystrophy. *JAMA Ophthalmol.* 131 807–810. 10.1001/jamaophthalmol.2013.2089 23599131

[B14] Daniele L. L. Caughey J. Volland S. Sharp R. C. Dhingra A. Williams D. S. (2019). Peroxisome turnover and diurnal modulation of antioxidant activity in retinal pigment epithelia utilizes microtubule-associated protein 1 light chain 3B (LC3B). *Am. J. Physiol. Cell Physiol.* 317 C1194–C1204. 10.1152/ajpcell.00185.2019 31577510 PMC6962520

[B15] Das Y. Baes M. (2019). Peroxisomal disorders and retinal degeneration. *Adv. Exp. Med. Biol.* 1185 317–321. 10.1007/978-3-030-27378-1_52 31884631

[B16] Das Y. Roose N. De Groef L. Fransen M. Moons L. Van Veldhoven P. P. (2019). Differential distribution of peroxisomal proteins points to specific roles of peroxisomes in the murine retina. *Mol. Cell. Biochem.* 456 53–62. 10.1007/s11010-018-3489-3 30604065

[B17] Edgar R. Domrachev M. Lash A. E. (2002). Gene expression omnibus: NCBI gene expression and hybridization array data repository. *Nucleic Acids Res.* 30 207–210. 10.1093/nar/30.1.207 11752295 PMC99122

[B18] Ferdinandusse S. Denis S. Mooyer P. A. Dekker C. Duran M. Soorani-Lunsing R. J. (2006). Clinical and biochemical spectrum of D-bifunctional protein deficiency. *Ann. Neurol.* 59 92–104. 10.1002/ana.20702 16278854

[B19] Fliesler S. J. Anderson R. E. (1983). Chemistry and metabolism of lipids in the vertebrate retina. *Prog. Lipid Res.* 22 79–131. 10.1016/0163-7827(83)90004-8 6348799

[B20] Folz S. J. Trobe J. D. (1991). The peroxisome and the eye. *Surv. Ophthalmol.* 35 353–368. 10.1016/0039-6257(91)90185-i 1710072

[B21] Glasgow B. J. Brown H. H. Hannah J. B. Foos R. Y. (1987). Ocular pathologic findings in neonatal adrenoleukodystrophy. *Ophthalmology* 94 1054–1060. 10.1016/s0161-6420(87)33345-7 3658367

[B22] Humphries M. M. Rancourt D. Farrar G. J. Kenna P. Hazel M. Bush R. A. (1997). Retinopathy induced in mice by targeted disruption of the rhodopsin gene. *Nat. Genet.* 15 216–219. 10.1038/ng0297-216 9020854

[B23] Huyghe S. Schmalbruch H. Hulshagen L. Van Veldhoven P. P. Baes M. Hartmann D. (2006). Peroxisomal multifunctional protein-2 deficiency causes motor deficits and glial lesions in the adult central nervous system. *Am. J. Pathol.* 168 1321–1334. 10.2353/ajpath.2006.041220 16565505 PMC1606565

[B24] Jaspers Y. R. J. Ferdinandusse S. Dijkstra I. M. E. Barendsen R. W. van Lenthe H. Kulik W. (2020). Comparison of the diagnostic performance of C26:0-lysophosphatidylcholine and very long-chain fatty acids analysis for peroxisomal disorders. *Front. Cell Dev. Biol.* 8:690. 10.3389/fcell.2020.00690 32903870 PMC7438929

[B25] Jun B. Mukherjee P. K. Asatryan A. Kautzmann M. A. Heap J. Gordon W. C. (2017). Elovanoids are novel cell-specific lipid mediators necessary for neuroprotective signaling for photoreceptor cell integrity. *Sci. Rep.* 7:5279. 10.1038/s41598-017-05433-7 28706274 PMC5509689

[B26] Kanan Y. Gordon W. C. Mukherjee P. K. Bazan N. G. Al-Ubaidi M. R. (2015). Neuroprotectin D1 is synthesized in the cone photoreceptor cell line 661W and elicits protection against light-induced stress. *Cell. Mol. Neurobiol.* 35 197–204. 10.1007/s10571-014-0111-4 25212825 PMC4381923

[B27] Kinoshita J. Peachey N. S. (2018). Noninvasive electroretinographic procedures for the study of the mouse retina. *Curr. Protoc. Mouse Biol.* 8 1–16. 10.1002/cpmo.39 30040236 PMC6060644

[B28] Lem J. Krasnoperova N. V. Clavert P. D. Kosaras B. Cameron D. A. Nicolò M. (1999). Morphological, physiological, and biochemical changes in rhodopsin knockout mice. *Proc. Natl. Acad. Sci. U.S.A.* 96 736–741. 10.1073/pnas.96.2.736 9892703 PMC15206

[B29] Liang Y. Fotiadis D. Maeda T. Maeda A. Modzelewska A. Filipek S. (2004). Rhodopsin signaling and organization in heterozygote rhodopsin knockout mice. *J. Biol. Chem.* 279 48189–48196. 10.1074/jbc.M408362200 15337746 PMC1351248

[B30] Lines M. A. Jobling R. Brady L. Marshall C. R. Scherer S. W. Rodriguez A. R. (2014). Peroxisomal D-bifunctional protein deficiency: three adults diagnosed by whole-exome sequencing. *Neurology* 82 963–968. 10.1212/wnl.0000000000000219 24553428 PMC3963001

[B31] Lobanova E. S. Schuhmann K. Finkelstein S. Lewis T. R. Cady M. A. Hao Y. (2019). Disrupted blood-retina lysophosphatidylcholine transport impairs photoreceptor health but not visual signal transduction. *J. Neurosci.* 39 9689–9701. 10.1523/jneurosci.1142-19.2019 31676603 PMC6891062

[B32] Martinez M. (1992). Abnormal profiles of polyunsaturated fatty acids in the brain, liver, kidney and retina of patients with peroxisomal disorders. *Brain Res.* 583 171–182. 10.1016/s0006-8993(10)80021-6 1504825

[B33] McMillan H. J. Worthylake T. Schwartzentruber J. Gottlieb C. C. Lawrence S. E. Mackenzie A. (2012). Specific combination of compound heterozygous mutations in 17beta-hydroxysteroid dehydrogenase type 4 (HSD17B4) defines a new subtype of D-bifunctional protein deficiency. *Orphanet J. Rare Dis.* 7:90. 10.1186/1750-1172-7-90 23181892 PMC3551712

[B34] Medema S. Mocking R. J. Koeter M. W. Vaz F. M. Meijer C. de Haan L. (2016). Levels of red blood cell fatty acids in patients with psychosis, their unaffected siblings, and healthy controls. *Schizophr. Bull.* 42 358–368. 10.1093/schbul/sbv133 26385764 PMC4753602

[B35] Mukherjee P. K. Marcheselli V. L. Serhan C. N. Bazan N. G. (2004). Neuroprotectin D1: a docosahexaenoic acid-derived docosatriene protects human retinal pigment epithelial cells from oxidative stress. *Proc. Natl. Acad. Sci. U.S.A.* 101 8491–8496. 10.1073/pnas.0402531101 15152078 PMC420421

[B36] Noguer M. T. Martinez M. (2010). Visual follow-up in peroxisomal-disorder patients treated with docosahexaenoic acid ethyl ester. *Invest. Ophthalmol. Vis. Sci.* 51 2277–2285. 10.1167/iovs.09-4020 19933185

[B37] Paker A. M. Sunness J. S. Brereton N. H. Speedie L. J. Albanna L. Dharmaraj S. (2010). Docosahexaenoic acid therapy in peroxisomal diseases: results of a double-blind, randomized trial. *Neurology* 75 826–830. 10.1212/WNL.0b013e3181f07061 20805528 PMC3013498

[B38] Price B. A. Sandoval I. M. Chan F. Nichols R. Roman-Sanchez R. Wensel T. G. (2012). Rhodopsin gene expression determines rod outer segment size and rod cell resistance to a dominant-negative neurodegeneration mutant. *PLoS One* 7:e49889. 10.1371/journal.pone.0049889 23185477 PMC3503812

[B39] Prusky G. T. Alam N. M. Beekman S. Douglas R. M. (2004). Rapid quantification of adult and developing mouse spatial vision using a virtual optomotor system. *Invest. Ophthalmol. Vis. Sci.* 45 4611–4616. 10.1167/iovs.04-0541 15557474

[B40] Reyes-Reveles J. Dhingra A. Alexander D. Bragin A. Philp N. J. Boesze-Battaglia K. (2017). Phagocytosis-dependent ketogenesis in retinal pigment epithelium. *J. Biol. Chem.* 292 8038–8047. 10.1074/jbc.M116.770784 28302729 PMC5427279

[B41] Rice D. S. Calandria J. M. Gordon W. C. Jun B. Zhou Y. Gelfman C. M. (2015). Adiponectin receptor 1 conserves docosahexaenoic acid and promotes photoreceptor cell survival. *Nat. Commun.* 6:6228. 10.1038/ncomms7228 25736573 PMC4351799

[B42] Rotstein N. P. Pennacchiotti G. L. Sprecher H. Aveldano M. I. (1996). Active synthesis of C24:5, n-3 fatty acid in retina. *Biochem. J.* 316 859–864. 10.1042/bj3160859 8670163 PMC1217429

[B43] Scott B. L. Bazan N. G. (1989). Membrane docosahexaenoate is supplied to the developing brain and retina by the liver. *Proc. Natl. Acad. Sci. U.S.A.* 86 2903–2907. 10.1073/pnas.86.8.2903 2523075 PMC287028

[B44] Shindou H. Koso H. Sasaki J. Nakanishi H. Sagara H. Nakagawa K. M. (2017). Docosahexaenoic acid preserves visual function by maintaining correct disc morphology in retinal photoreceptor cells. *J. Biol. Chem.* 292 12054–12064. 10.1074/jbc.M117.790568 28578316 PMC5519357

[B45] Simon M. V. Agnolazza D. L. German O. L. Garelli A. Politi L. E. Agbaga M. P. (2016). Synthesis of docosahexaenoic acid from eicosapentaenoic acid in retina neurons protects photoreceptors from oxidative stress. *J. Neurochem.* 136 931–946. 10.1111/jnc.13487 26662863 PMC4755815

[B46] Sluch V. M. Banks A. Li H. Crowley M. A. Davis V. Xiang C. (2018). ADIPOR1 is essential for vision and its RPE expression is lost in the Mfrp. *Sci. Rep.* 8:14339. 10.1038/s41598-018-32579-9 30254279 PMC6156493

[B47] Smith C. E. Poulter J. A. Levin A. V. Capasso J. E. Price S. Ben-Yosef T. (2016). Spectrum of PEX1 and PEX6 variants in Heimler syndrome. *Eur. J. Hum. Genet.* 24 1565–1571. 10.1038/ejhg.2016.62 27302843 PMC5026821

[B48] Strauss O. (2005). The retinal pigment epithelium in visual function. *Physiol. Rev.* 85 845–881. 10.1152/physrev.00021.2004 15987797

[B49] Swaroop A. Kim D. Forrest D. (2010). Transcriptional regulation of photoreceptor development and homeostasis in the mammalian retina. *Nat. Rev. Neurosci.* 11 563–576. 10.1038/nrn2880 20648062 PMC11346175

[B50] Taverna F. Goveia J. Karakach T. K. Khan S. Rohlenova K. Treps L. (2020). BIOMEX: an interactive workflow for (single cell) omics data interpretation and visualization. *Nucleic Acids Res.* 48 W385–W394. 10.1093/nar/gkaa332 32392297 PMC7319461

[B51] Tinoco J. (1982). Dietary requirements and functions of alpha-linolenic acid in animals. *Prog. Lipid Res.* 21 1–45. 10.1016/0163-7827(82)90015-7 6287500

[B52] Van Veldhoven P. P. (2010). Biochemistry and genetics of inherited disorders of peroxisomal fatty acid metabolism. *J. Lipid Res.* 51 2863–2895. 10.1194/jlr.R005959 20558530 PMC2936746

[B53] Vaz F. M. McDermott J. H. Alders M. Wortmann S. B. Kölker S. Pras-Raves M. L. (2019). Mutations in PCYT2 disrupt etherlipid biosynthesis and cause a complex hereditary spastic paraplegia. *Brain* 142 3382–3397. 10.1093/brain/awz291 31637422 PMC6821184

[B54] Wanders R. J. Waterham H. R. (2006). Biochemistry of mammalian peroxisomes revisited. *Annu. Rev. Biochem.* 75 295–332. 10.1146/annurev.biochem.74.082803.133329 16756494

[B55] Wang N. Anderson R. E. (1993). Synthesis of docosahexaenoic acid by retina and retinal pigment epithelium. *Biochemistry* 32 13703–13709. 10.1021/bi00212a040 7903049

[B56] Waterham H. R. Ferdinandusse S. Wanders R. J. (2016). Human disorders of peroxisome metabolism and biogenesis. *Biochim. Biophys. Acta* 1863 922–933. 10.1016/j.bbamcr.2015.11.015 26611709

[B57] Wong B. H. Chan J. P. Cazenave-Gassiot A. Poh R. W. Foo J. C. Galam D. L. (2016). Mfsd2a is a transporter for the essential omega-3 fatty acid docosahexaenoic acid (DHA) in eye and is important for photoreceptor cell development. *J. Biol. Chem.* 291 10501–10514. 10.1074/jbc.M116.721340 27008858 PMC4865901

[B58] Xin-Zhao Wang C. Zhang K. Aredo B. Lu H. Ufret-Vincenty R. L. (2012). Novel method for the rapid isolation of RPE cells specifically for RNA extraction and analysis. *Exp. Eye Res.* 102 1–9. 10.1016/j.exer.2012.06.003 22721721 PMC3432720

[B59] Zaki M. S. Heller R. Thoenes M. Nurnberg G. Stern-Schneider G. Nurnberg P. (2016). PEX6 is expressed in photoreceptor cilia and mutated in deafblindness with enamel dysplasia and microcephaly. *Hum. Mutat.* 37 170–174. 10.1002/humu.22934 26593283

